# ^18^F-florbetapir PET as a marker of myelin integrity across the Alzheimer’s disease spectrum

**DOI:** 10.1007/s00259-021-05493-y

**Published:** 2021-09-28

**Authors:** Alexis Moscoso, Jesús Silva-Rodríguez, Jose Manuel Aldrey, Julia Cortés, Juan Manuel Pías-Peleteiro, Álvaro Ruibal, Pablo Aguiar, for the Alzheimer’s Disease Neuroimaging Initiative

**Affiliations:** 1 Nuclear Medicine Department and Molecular Imaging Group, University Hospital Santiago de Compostela, IDIS, Travesía da Choupana S/N, 15706 Santiago de Compostela, Spain; 2 Department of Psychiatry and Neurochemistry, Institute of Neuroscience and Physiology, The Sahlgrenska Academy, and The Wallenberg Centre for Molecular and Translational Medicine, University of Gothenburg, Gothenburg, Sweden; 3 Neurology Department, University Hospital Santiago de Compostela, Travesía da Choupana S/N, 15706 Santiago de Compostela, Spain; 4 Molecular Imaging Group, Department of Radiology, CIMUS, University of Santiago de Compostela (USC), Campus Vida, 15782 Santiago de Compostela, Spain

**Keywords:** ^18^F-florbetapir, Alzheimer, Myelin, Progression, White matter

## Abstract

**Purpose:**

Recent evidence suggests that PET imaging with amyloid-β (Aβ) tracers can be used to assess myelin integrity in cerebral white matter (WM). Alzheimer’s disease (AD) is characterized by myelin changes that are believed to occur early in the disease course. Nevertheless, the extent to which demyelination, as measured with Aβ PET, contributes to AD progression remains unexplored.

**Methods:**

Participants with concurrent ^18^F-florbetapir (FBP) PET, MRI, and cerebrospinal fluid (CSF) examinations were included (241 cognitively normal, 347 Aβ-positive cognitively impaired, and 207 Aβ-negative cognitively impaired subjects). A subset of these participants had also available diffusion tensor imaging (DTI) images (*n* = 195). We investigated cross-sectional associations of FBP retention in the white matter (WM) with MRI-based markers of WM degeneration, AD clinical progression, and fluid biomarkers. In longitudinal analyses, we used linear mixed models to assess whether FBP retention in normal-appearing WM (NAWM) predicted progression of WM hyperintensity (WMH) burden and clinical decline.

**Results:**

In AD-continuum individuals, FBP retention in NAWM was (1) higher compared with WMH regions, (2) associated with DTI-based measures of WM integrity, and (3) associated with longitudinal progression of WMH burden. FBP uptake in WM decreased across the AD continuum and with increasingly abnormal CSF biomarkers of AD. Furthermore, FBP retention in the WM was associated with large-calibre axon degeneration as reflected by abnormal plasma neurofilament light chain levels. Low FBP uptake in NAWM predicted clinical decline in preclinical and prodromal AD, independent of demographics, global cortical Aβ, and WMH burden. Most of these associations were also observed in Aβ-negative cognitively impaired individuals.

**Conclusion:**

These results support the hypothesis that FBP retention in the WM is myelin-related. Demyelination levels progressed across the AD continuum and were associated with clinical progression at early stages, suggesting that this pathologic process might be a relevant degenerative feature in the disease course.

**Supplementary Information:**

The online version contains supplementary material available at 10.1007/s00259-021-05493-y.

## Introduction

Over the past decades, Alzheimer’s disease (AD) has been conceptualized as a grey matter pathology characterized by the accumulation of amyloid-β (Aβ) plaques and neurofibrillary tangles [1, 2]. Nevertheless, numerous neuropathological and neuroimaging studies indicate that white matter (WM) abnormalities are also commonly present among AD patients [3–8]. While these findings have been traditionally interpreted as comorbidities, accumulating evidence from our group [9] and others [10–14] points towards a link between WM pathology and AD-specific neuropathologic changes, suggesting that WM degeneration is a characteristic feature of the AD pathological cascade. Among these pathological changes, one of the most prominent is the generalized degradation of axonal myelin sheets in cerebral WM [15–17]. These myelin alterations have been found to be related to cortical Aβ deposition in the preclinical stage of AD [18], indicating that WM alterations might reflect early neurodegenerative processes in the AD pathological course.

Supported by recent investigations, Aβ PET has been recently repurposed as a marker of myelin integrity in cerebral WM [19–26]. Although the binding mechanism is currently not fully understood, the elevated WM signal observed with Aβ PET tracers might be explained by the affinity of the later for the beta-sheet structure displayed by the myelin basic protein [27]. Aβ PET uptake in the WM has been proven sensitive enough to track myelin changes in multiple sclerosis [20], even in radiologically normal-appearing WM (NAWM) [28, 29]. Nevertheless, the aforementioned results have been only reported in multiple sclerosis and aging, and the association of demyelination, as measured with Aβ PET, with pathologic features and progression in AD has not yet been investigated.

In this study, we hypothesized that regional ^18^F-florbetapir (FBP) retention in the WM, an established Aβ PET radiotracer, reflects local myelin integrity, and that such myelin alterations are associated with AD pathology and progression. To test our hypotheses, we examined a large and well-phenotyped cohort of participants in the Alzheimer’s Disease Neuroimaging Initiative (ADNI) that included normal aging subjects as well as preclinical, prodromal, mild AD, and non-AD amnestic patients with available imaging and fluid biomarker data. Specifically, we investigated (1) whether FBP retention in the WM correlates with MRI-based measures of WM degeneration, assessed with Fluid attenuated inversion recovery (FLAIR) and diffusion tensor imaging (DTI); (2) how FBP retention in the WM changes across the AD continuum and with core biomarkers of AD, as well as in non-AD amnestic patients; and (3) whether FBP retention in the WM is a predictor of longitudinal cognitive decline.

## Methods

### Participants

ADNI participants who underwent concurrent Aβ PET, T1-weighted and FLAIR MRI, and cerebrospinal fluid (CSF) examinations were considered in this study. See Supplementary Methods 1 for a description of the ADNI study. Participants were further stratified by Aβ status (A±) using standardized uptake value ratio (SUVR) values computed by the ADNI PET core and reported in the “SUMMARYSUVR_WHOLECEREBNORM_1.11CUTOFF” field of the “UCBERKELEYAV45_05_12_20” CSV file [30, 31] and in cognitively normal (CN), mild cognitive impairment (MCI), and AD dementia. Detailed information about the eligibility criteria for the different diagnostic cohorts can be found at http://adni.loni.usc.edu/methods/documents/. Since we are interested in the role of demyelination across the AD continuum, our primary analysis was focused on individuals in the Alzheimer’s continuum, i.e. those with cognitive impairment (MCI and AD) and a positive Aβ status (A+) as well as those cognitively unimpaired (both A- and A+). This resulted in a primary study cohort of 588 subjects. A subset of these participants had also DTI scans available at baseline (*n* = 151). At least one longitudinal FLAIR scan was available in 493 study participants (mean follow-up time 2.1 years). Demographics and biomarker information of the study cohort are summarized in Table 1. In a secondary analysis, we also investigated non-AD amnestic participants, which included A- MCI (*n* = 192) and A- AD dementia (*n* = 15). Forty-four of these participants had available baseline DTI scans, and at least one longitudinal FLAIR scan was available in 203 participants (mean follow-up time 2.5 years). Demographics and biomarker information of this secondary study cohort are reported in Supplementary Table 1. All participants provided written informed consent approved by the institutional review board of each ADNI participating institution.

**Table 1:** Demographic and biomarker characteristics of the study participants

	A- CN	A + CN	A + MCI	A + AD
Age, y	72.2 (6.0)	75.4 (5.9)	73.1 (6.8)	74.1 (8.4)
Women, n, %	79, 47%	52, 70%	103, 43%	49, 45%
APOE ε4 carriers, n, %	34, 20%	35, 47%	159, 67%	84, 76%
Education, y	17 (12–20)	16 (12–20)	16 (9–20)	16 (9–20)
ADAS-Cog 13	8.5 (4.3)	9.5 (4.3)	17.1 (6.9)	31.6 (8.5)
CSF Aβ-42, pg/ml	1596 (351–3462)	781 (203–2717)	711 (267–2345)	583 (256–1694)
CSF p-tau181, pg/ml	17.8 (8.0–50.8)	24.2 (10.3–60.0)	29.5 (10.2–92.1)	34.4 (14.8–83.3)
Plasma NfL, pg/ml	29.2 (8.0–282.2)	36.0 (14.1–74.5)	38.6 (12.0–124.4)	42.4 (19.8–138.9)
WMH volume, cm^3^	0.2 (0–26.0)	1.3 (0–44.7)	0.9 (0–27.6)	1.7 (0–25.6)

Age and cognitive data (ADAS-Cog 13) were reported as mean (standard deviation). Education years, fluid biomarker levels, and white matter hyperintensity volume were reported as median (range)

### Magnetic resonance imaging

MRI acquisition and pre-processing protocols in ADNI are described in detail elsewhere [32]. All included subjects were scanned in 3T MRI devices. Structural T1 images were normalized to Montreal Neurological Institute (MNI) space and segmented using Statistical Parametric Mapping 12 (SPM12, Wellcome Department of Clinical Neurology, London, UK). Baseline and longitudinal FLAIR scans were processed using the Lesion Segmentation Toolbox [33] in SPM12 as previously described [9, 33] to derive binary WM hyperintensity (WMH) masks.

### ^18^F-florbetapir PET

FBP PET scans were acquired and pre-processed following the ADNI pipeline [34] (http://adni.loni.usc.edu/methods/pet-analysis-method/pet-analysis/). For quantification of FBP retention in the WM, we followed the same approach used in [35]. Briefly, FBP images were first coregistered to the corresponding T1 scan using SPM12. A WM mask containing both NAWM and WMH was then generated by merging the binarized WM segment from SPM12 (generated after thresholding the WM probability map with 0.5) and the binary WMH mask previously generated from FLAIR scans. Note that by combining both segmentations, we avoid incorrect classification of WM hypointense areas in the T1 scan as CSF or grey matter [28]. The inverse of the deformation field from spatial normalization was then used to propagate a predefined cerebral mask from MNI space into each individual’s native space, and this mask was intersected with the previously derived WM mask to obtain a cerebral WM mask (i.e. excluding the brainstem and cerebellar WM). To minimize partial volume effects, we (1) excluded small WMH clusters with fewer than 27 voxels (~ 27 mm3, corresponding to a 3-mm isotropic voxel) from the analysis to minimize contamination due to spill-in counts from surrounding NAWM, and (2) we eroded the resulting WM mask so that voxels within 2-mm distance from any non-WM voxel were excluded [28]. Finally, SUVR was calculated in NAWM and WMH regions by using the cerebellar grey matter as the reference region. While recent works point that the whole cerebellum [36] or the WM [37] might be superior reference regions, we choose the traditional cerebellar grey matter [38] to avoid any potential circular analysis due to the inclusion of cerebellar WM.

For voxel-wise analyses, coregistered FBP PET scans were spatially normalized to MNI space using the deformation fields obtained from the T1 spatial normalization, masked with a binary WM mask defined in MNI space, and smoothed using an 8-mm isotropic filter.

To statistically control analyses for continuous levels of Aβ burden within A + and A- subjects, we also measured continuous SUVR (using cerebellar grey matter as reference region) within the ADNI ROI that was previously used for establishing Aβ status [30].

### Fluid biomarkers

CSF levels of Aβ_1-42_ (Aβ-42) and phosphorylated tau181 (p-tau181) were measured using a fully automated Roche Elecsys electrochemiluminescence immunoassay [39]. Plasma levels of neurofilament light chain (NfL), a marker of subcortical large-calibre axonal degeneration [40, 41], were measured from blood samples as described previously [42]. A detailed description of CSF and blood sampling procedures can be found at http://adni.loni.usc.edu/methods/.

### Diffusion tensor imaging

Diffusion-weighted images were acquired in a subset of participants (those scanned with GE scanners). Acquisition protocols, as well as pre-processing and post-processing steps, were described in detail in previous studies [43]. Here, we used fractional anisotropy (FA) images provided by ADNI to quantify WM integrity [44] in NAWM and WMH. For this, FA images were coregistered to the corresponding T1 scan using SPM12. The results were visually inspected to ascertain a correct registration. Mean FA in NAWM and WMH was measured within the eroded WM mask defined above, after excluding voxels with FA < 0.25 to exclude spurious fibre tracts [45].

### Clinical assessments

Global cognitive performance was assessed with the Alzheimer’s Disease Assessment Scale—Cognitive 13-Item (ADAS-Cog 13) at baseline and in subsequent annual follow-up visits. Changes in clinical diagnosis at follow-up were determined by a consensus committee. Further details can be found at http://adni.loni.usc.edu/methods/.

### Statistical analysis

We first investigated how FBP retention in the WM correlates with cross-sectional MRI markers of WM degeneration. For this, we (1) compared FBP SUVR in NAWM and WMH using paired *t* tests and (2) fitted linear models to assess cross-sectional associations between average FBP SUVR and FA in these regions. Linear models were adjusted for age, sex, and clinical diagnosis. Furthermore, since Aβ PET tracer retention in the WM has been found to be positively correlated with global tracer uptake in the cortex [28, 46, 47], which likely reflects partial volume effects but also binding to diffuse plaques and cerebrovascular amyloid angiopathy [46, 48, 49], we also regressed-out this confounding effect in order to isolate myelin binding contributions to FBP SUVRs. For this, we used the entire CN cohort to fit a linear model describing the dependence of NAWM and WMH SUVR with cortical FBP SUVR (see Supplementary Methods 2). After regressing-out cortical FBP dependence, we transformed the resulting variables to z-scores using CN levels as reference. We referred to this adjusted measure as “adjusted NAWM (or adjusted WMH) SUVR” in the present and subsequent analyses.

We also investigated whether low FBP uptake in NAWM is an early marker of WM damage in longitudinal analyses. Linear mixed models with subject-specific intercepts, adjusted for age, sex, and clinical diagnosis, were fitted to assess whether adjusted NAWM SUVR was a predictor of faster WMH accumulation.

Next, we investigated how FBP retention in the WM changes across the AD continuum. Voxel-wise analyses adjusted for age, sex, and global cortical FBP SUVR were conducted in the WM to assess group-level differences in FBP SUVR across the preclinical, prodromal, and dementia stages of AD. The A- CN sample was used as the reference group, and statistical maps were thresholded using *p*_FDR_ < 0.001. We also conducted ROI-level analyses comparing adjusted SUVR in NAWM and WMH across AD stages. In addition, we investigated the diagnostic added value of these two measures for identifying advanced disease stages in AD. For this, we analysed the incremental discriminative accuracy provided by adjusted NAWM and WMH SUVR to classify A+ CN vs A+ MCI and A+ CN vs A+ AD. The areas under the ROC curve (AUC) of two logistic regression models, one including cortical SUVR as a predictor and the second including both cortical SUVR and adjusted NAWM or WMH SUVR, were compared using a bootstrap procedure. Both models included age and sex terms as covariates. In addition, in the smaller subset with available DTI scans, we also performed a preliminary head-to-head comparison of the discriminative accuracy provided by the latter models in comparison to a model based on FA, cortical SUVR, and covariates.

We then investigated the associations of FBP retention in NAWM and WMH with fluid biomarker levels. Linear regressions adjusted for age, sex, clinical diagnosis, and cortical FBP SUVR were separately fitted in A- and A + subjects to assess the relationship between fluid biomarkers and adjusted SUVR in NAWM and WMH. Fluid biomarker levels were log-transformed to reduce the skewness of model residuals.

Finally, we used linear mixed effects models with subject-specific random intercepts to investigate whether demyelination in NAWM, as reflected by low adjusted SUVR in this region, is associated with accelerated rates of longitudinal cognitive decline. The models were adjusted for age, sex, and years of education, as well as for global cortical FBP SUVR and WMH volume given their known associations with longitudinal cognitive deterioration [50, 51]. Cox regression, adjusted for the same covariates, was used to test whether adjusted SUVR in NAWM was associated with increased risk of progression to MCI or dementia. These associations were also tested using dichotomized versions of adjusted NAWM SUVR; the cut-point was defined as the adjusted NAWM SUVR that results in 90% sensitivity for the identification of A+ AD dementia participants [52, 53], yielding an adjusted NAWM SUVR cut-point of − 0.57.

Following guidelines from the statistical literature that do not recommend the use of multiple comparisons correction for hypothesis-driven studies with a limited number of planned comparisons [54], we did not perform multiple comparisons correction except for voxel-wise analyses.

## Results

### FBP retention in the WM and MRI markers of WM degeneration

**Fig. 1:**
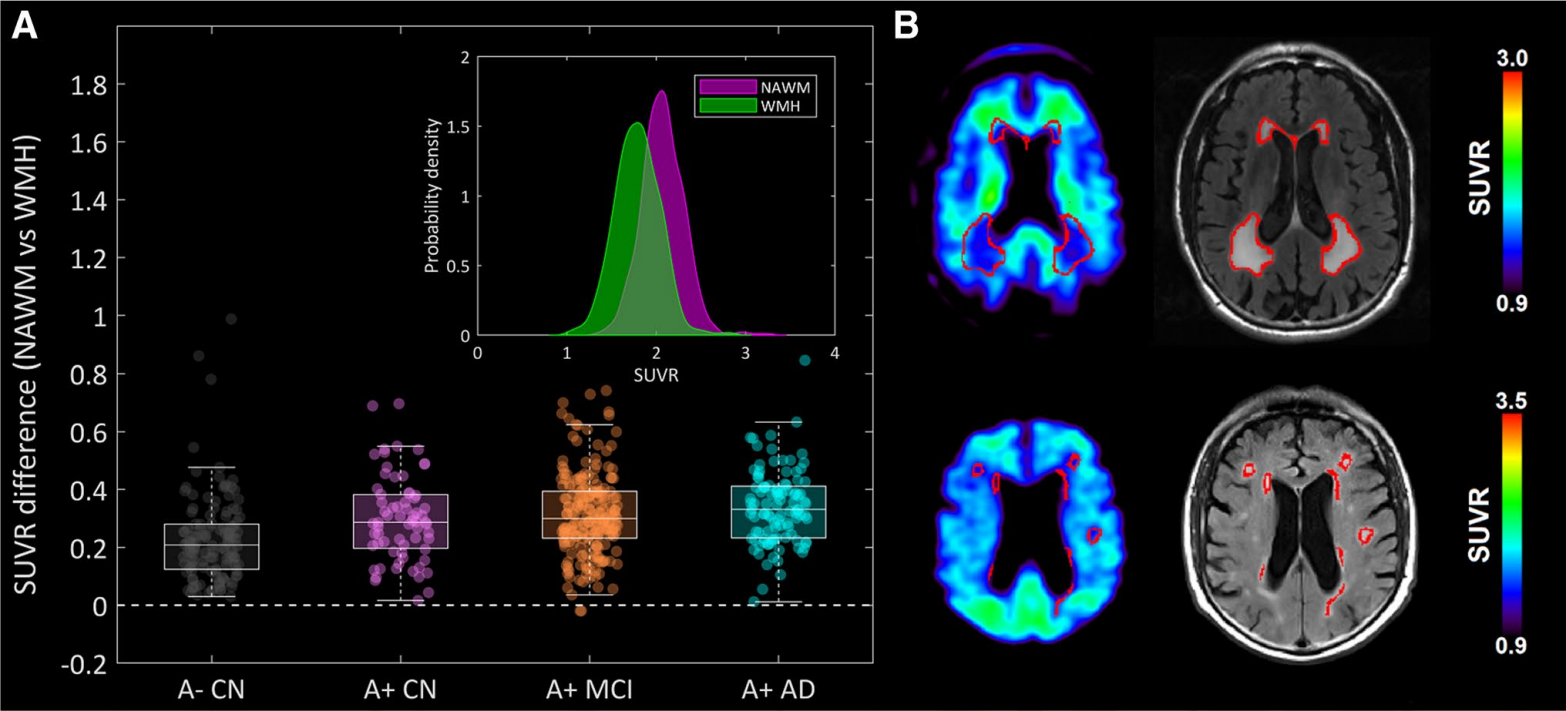
Reduced FBP retention in WMH compared to NAWM. (**A**) SUVR difference between NAWM and WMH, across the different AD stages. All the groups showed higher NAWM SUVR (*p* < 0.001). (**B**) Examples of two study participants showing reduced FBP retention in WMH

We first investigated associations between FBP retention in the WM and MRI-based measures of WM degeneration that encompass demyelination. After the erosion procedure, 421 subjects demonstrated at least some WMH regions. Consistent with the notion that FBP retention in the WM reflects myelin integrity, we observed that, compared with NAWM, WMH regions showed lower FBP SUVRs across the different AD stages (Fig. 1A, median difference 0.25–0.35, *p* < 0.001 for all tests). Examples of two representative study participants showing reduced FBP retention in WMH can be seen in Fig. 1B. Similar findings were obtained in the secondary cohort of A- cognitively impaired individuals (Supplementary Fig. 1). Furthermore, lower adjusted FBP SUVR was associated with lower WM integrity as reflected by lower FA in NAWM (β = 0.21, *p* = 0.04) but not in WMH (β =  − 0.08, *p* = 0.53) (Supplementary Fig. 2). This association, however, was not found in the A- cognitively impaired group (NAWM: β = 0.04, *p* = 0.79, WMH: β =  − 0.19, *p* = 0.28).

**Fig. 2:**
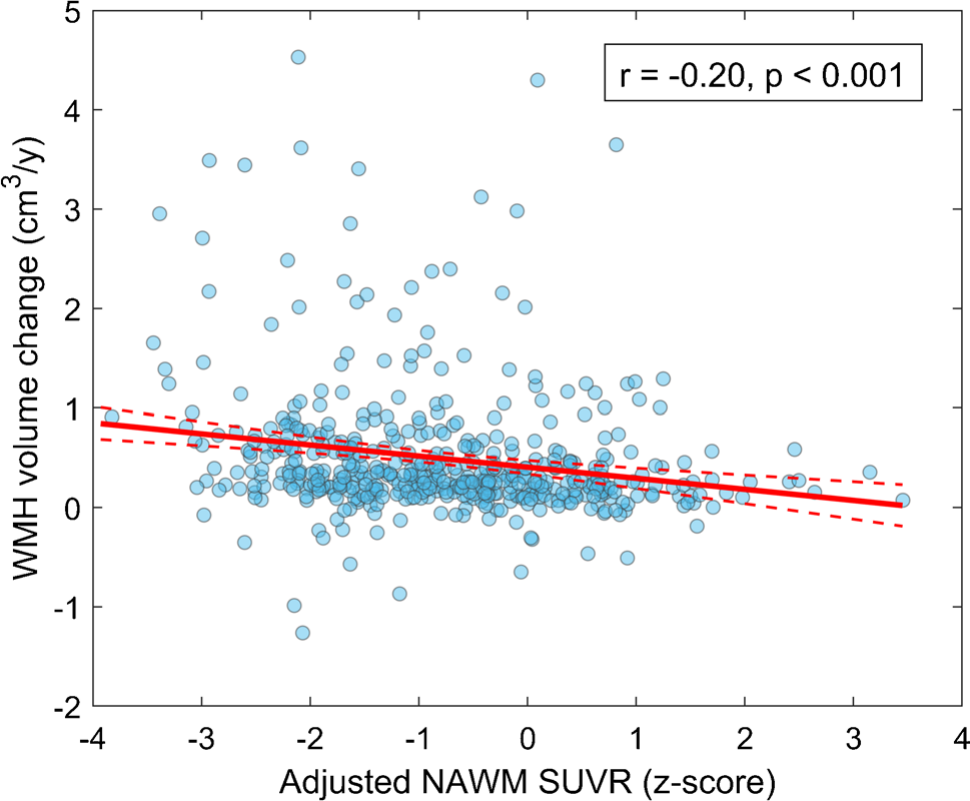
Association between baseline adjusted FBP SUVR in NAWM and longitudinal WMH volume change. Red solid line depicts the regression line. Dashed lines represent 95% confidence intervals. We reported the (unadjusted for covariates) Pearson correlation coefficient describing the correlation between these two variables

In analyses with longitudinal FLAIR scans, low baseline adjusted NAWM SUVR was associated with faster longitudinal increases in WMH volume (b =  − 0.13 cm^3^/z-score/y, *p* < 0.001; Fig. 2), i.e. this measure predicted the conversion of NAWM into WMH. Identical results were observed in the A- cognitively impaired cohort (b =  − 0.19, cm^3^/z-score/y, *p* < 0.001; Supplementary Fig. 3).

### FBP retention in the WM decreases across the AD spectrum

**Fig. 3:**
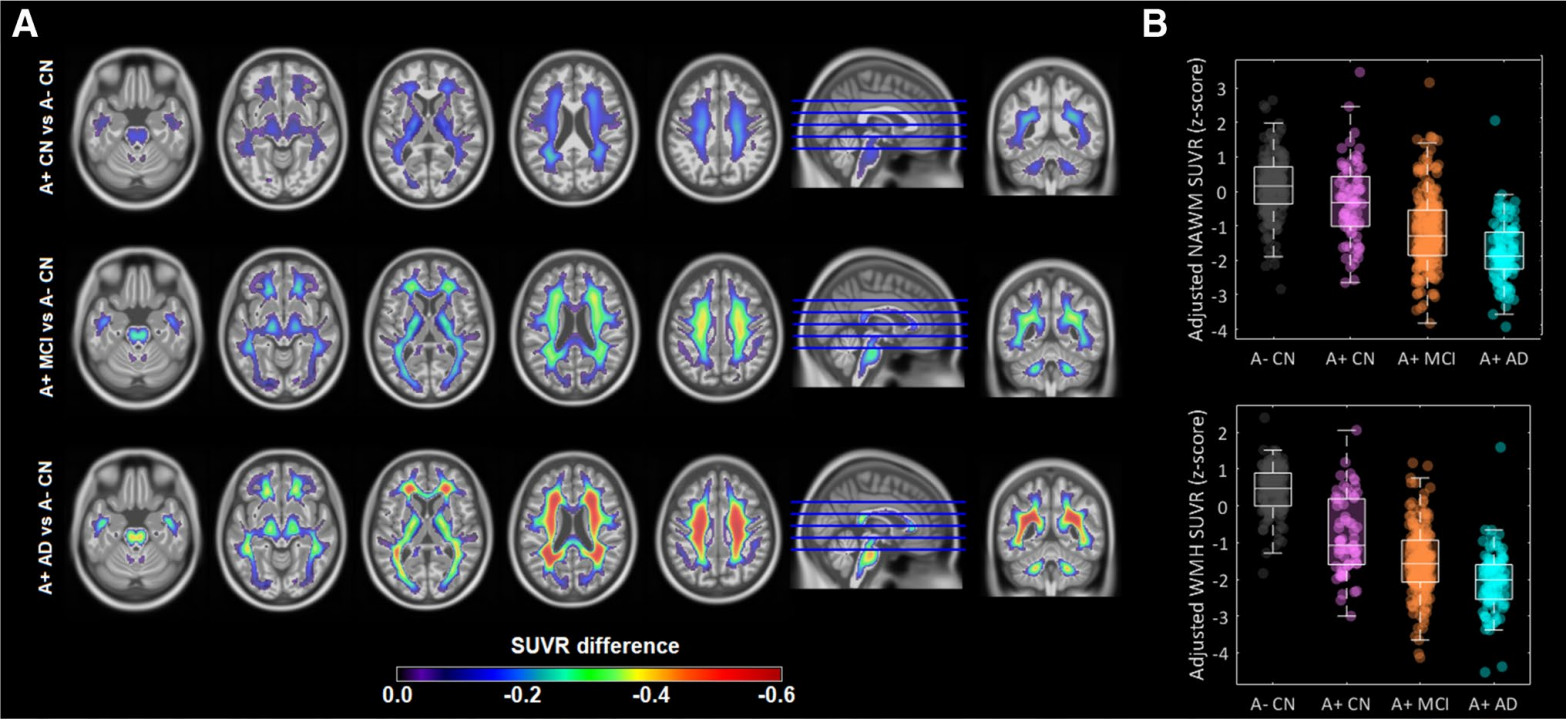
FBP retention in the WM across the AD spectrum. (**A**) Voxel-wise analyses, adjusted for age, sex, and cortical FBP SUVR, contrasting SUVR levels in the WM of the different AD stages (the A- CN cohort was used as the reference). Statistical maps were thresholded using a *p*_FDR_ < 0.001. (**B**) Boxplots describing group-level adjusted SUVR in NAWM and WMH

Next, we explored whether FBP-measured demyelination levels increase across the AD continuum. Voxel-wise analyses revealed extensive WM areas that showed reduced FBP SUVR, progressing in severity and spatial extension with increasing AD stages (Fig. 3A). Furthermore, the association patterns exceeded cerebral WM and further involved non-cerebral structures such as the brainstem and cerebellar WM in all AD stages. In analyses at the ROI level, we observed that adjusted SUVRs in both NAWM and WMH decreased with progressing AD stages (Fig. 3B, *p* < 0.001 for all consecutive comparisons). In a similar manner, demyelination increased with clinical severity in A- cognitively impaired individuals (Supplementary Fig. 4). Both adjusted NAWM and adjusted WMH SUVR significantly increased the discriminative accuracy of FBP PET to classify A+ CN vs A+ MCI and A+ CN vs A+ AD (Supplementary Fig. 5). Adjusted SUVRs outperformed FA measures in the discrimination of A+ CN vs A+ AD, but performances were slightly lower in A+ CN vs A+ MCI (Supplementary Table 2).

### Low FBP uptake in the WM correlates with abnormal CSF and plasma biomarkers

**Fig. 4:**
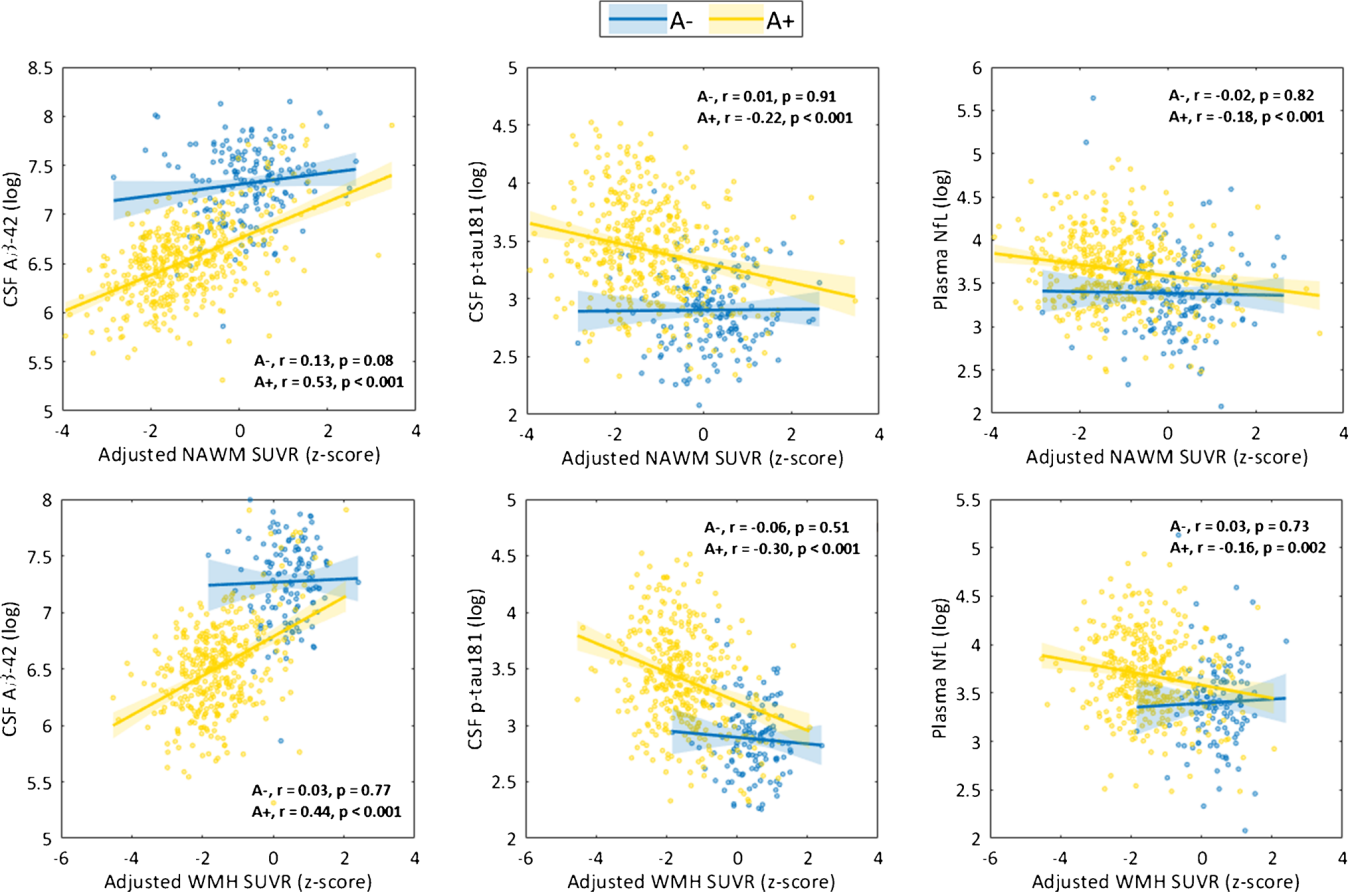
Associations of adjusted SUVR in NAWM and WMH with CSF and plasma biomarkers. Analyses were performed stratified by Aβ status. We reported the (unadjusted for covariates) Pearson correlation coefficient describing the correlation between the two represented variables

Consistent with previous section findings of progressive demyelination along the AD continuum, we found that low adjusted SUVR in both NAWM and WMH was associated with more pathological CSF Aβ-42 and p-tau181 levels only among subjects in the AD continuum (A+) (Fig. 4). These findings remain largely unchanged when adjusting for demographics and cortical FBP SUVR, indicating independent contributions of demyelination to pathological core AD biomarkers (Table 2). Furthermore, low adjusted SUVR was also associated with higher plasma NfL levels among A+ subjects (Fig. 4, right panels). When including covariates, this association, although attenuated, was still statistically significant for NAWM (Table 2). Among A- cognitively impaired individuals, only CSF Aβ-42 and plasma NfL showed significant associations with adjusted WM measures (Supplementary Fig. 6), though the association with plasma NfL was fully mediated by covariates (NAWM: CSF Aβ-42, *p*_adjusted_ < 0.001, plasma NfL, *p*_adjusted_ = 0.28; WMH: CSF Aβ-42, *p*_adjusted_ < 0.001).

**Table 2:** Linear model coefficients accounting for the effects of demographic factors, cortical FBP SUVR, and adjusted NAWM and WMH FBP SUVR on CSF and plasma biomarkers among A + individuals

	A +		
	CSF Aβ-42	CSF p-tau181	Plasma NfL
Model 1 coefficients			
Age	-0.02	0.00	0.46***
Female sex	0.36***	0.3**	0.20*
MCI	0.20	0.38**	0.21
AD	0.00	0.57***	0.39*
Cortical SUVR	-0.23***	0.23***	0.10*
Adjusted NAWM SUVR	0.52***	-0.15**	-0.13**
Model 2 coefficients			
Age	-0.01	0.03	0.46***
Female sex	0.37***	0.29**	0.20*
MCI	0.06	0.44**	0.27*
AD	-0.24	0.66**	0.49**
Cortical SUVR	0.05	0.16***	0.04
Adjusted WMH SUVR	0.42***	-0.12*	-0.07

Linear models including age, sex, clinical diagnosis, cortical FBP SUVR, and NAWM (Model 1) or WMH (Model 2) FBP SUVR as covariates were fitted to describe fluid biomarker levels (dependent variables). Model coefficients of continuous variables were reported as standardized βs. Categorical variable coefficients describe the change in z-score units of fluid biomarkers (references were CN and male sex)

^*^p < 0.05, **p < 0.01, ***p < *0.001*

### Low FBP retention in NAWM is associated with accelerated cognitive decline

**Fig. 5:**
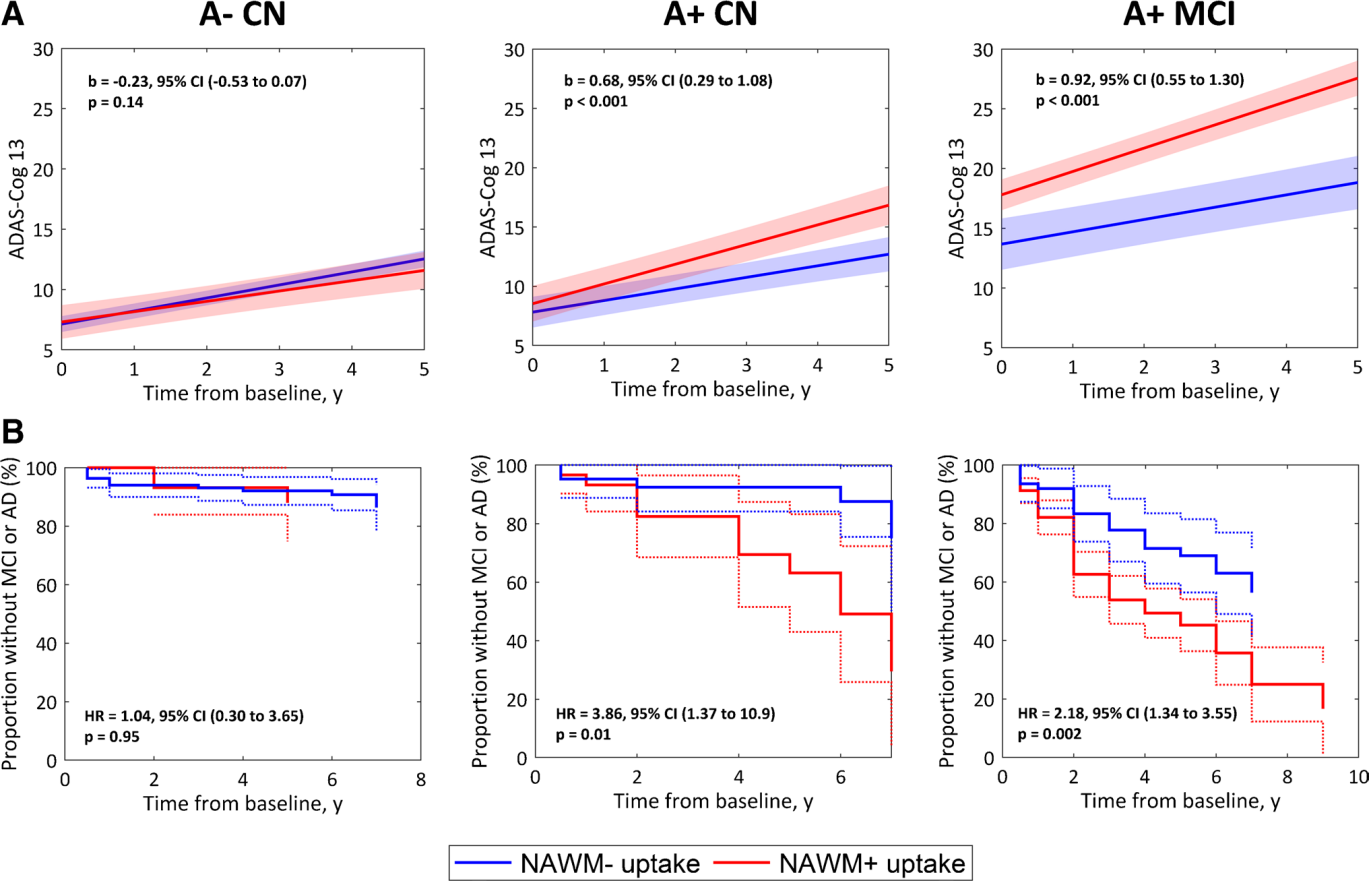
Associations between dichotomous adjusted FBP SUVR in NAWM and longitudinal clinical decline. (**A**) Estimated average trajectories of ADAS-Cog 13 for subjects with non-pathological NAWM uptake (NAWM-) (blue line) or pathological NAWM uptake (NAWM+) (red line), stratified in A- CN, preclinical AD, and prodromal AD groups. Group trajectories were estimated using covariate-unadjusted linear mixed models with subject-specific intercepts. Model coefficients (**B**) indicate the increase in annual rate of change of ADAS-Cog 13 in the NAWM+ uptake group compared to the NAWM- group. B) Kaplan–Meier survival curves describing the risk of progression to MCI (A- CN and A+ CN) or AD dementia (A+ MCI) for the NAWM- and NAWM+ uptake groups. HR represent covariate-unadjusted Cox-based hazard ratios

Finally, we investigated whether subtle demyelination changes, not captured by FLAIR imaging as WMH, accounted for cognitive changes across the AD spectrum and normal aging. For this, we tested how adjusted SUVRs in NAWM correlate with both cross-sectional and longitudinal cognitive decline. Cross-sectionally, low adjusted SUVR in NAWM was significantly associated with higher ADAS-Cog 13 scores only in A+ MCI participants (β =  − 0.25, *p* < 0.001, adjusted for age, sex, education years, cortical FBP SUVR, and WMH volume). Among A- cognitively impaired participants, only A- AD dementia participants showed an association between low adjusted FBP SUVR in NAWM and lower cognitive performance (A- MCI: β =  − 0.04, *p* = 0.58; A- AD dementia: β =  − 0.84, *p* = 0.018). Longitudinally, low adjusted SUVR in NAWM predicted faster cognitive decline in both A+ CN and A+ MCI participants, independent of demographics, cortical FBP SUVR, and WMH volume (Table 3). These results remained when using dichotomized adjusted NAWM SUVR (Fig. 5A). In the A- cognitively impaired group, low adjusted FBP SUVR in NAWM was associated with longitudinal cognitive decline only in the A- AD dementia group (A- MCI: β = 0.00, *p* = 0.94; A- AD dementia: β =  − 1.51, *p* < 0.001). Similar results were obtained when dichotomizing adjusted NAWM SUVR (Supplementary Fig. 7).

**Table 3:** Linear mixed model time-interaction coefficients accounting for the effects of demographics, WMH volume, cortical FBP SUVR, and adjusted NAWM FBP SUVR on longitudinal change in ADAS-Cog 13

	A- CN	A + CN	A + MCI
Coefficients			
Age × time	0.02	0.00	− 0.03*
Female sex × time	0.07**	0.03	0.02
Education × time	0.02	0.02	0.04*
WMH × time	0.01	0.00	0.01
Cortical SUVR × time	0.02	0.06*	0.09***
Adjusted NAWM SUVR × time	0.00	− 0.04*	− 0.07***

Linear mixed models included fixed effects of age, sex, education years, WMH volume, cortical FBP SUVR, and adjusted NAWM FBP SUVR, as well as their interactions with time. Coefficients represent the annual change in ADAS-Cog 13 (in baseline standard deviation units) with 1 standard deviation change of the baseline covariate.

^*^p < 0.05, **p < 0.01, ***p < *0.001*

In line with the previous results, survival analyses showed that low adjusted SUVR in NAWM was also associated with faster progression to AD dementia in A+ MCI subjects (b =  − 0.51, *p* < 0.001, adjusted for age, sex, education, WMH volume, and cortical FBP SUVR), but not with progression to MCI in A- CN (b = 0.11, *p* = 0.77) and A+ CN (b =  − 0.44, *p* = 0.13). When using dichotomous adjusted NAWM SUVR, we recapitulated the previous continuous association in A+ MCI but also found a statistically significant association in A+ CN individuals (Fig. 5B). Adjusted NAWM SUVR was not associated with increased risk of progression to dementia in the A- MCI group (HR = 1.38, 95% CI (0.18–3.09), *p* = 0.69).

## Discussion

In this study, we explored the associations of demyelination, as reflected by low FBP retention in cerebral WM, with MRI markers of WM integrity, AD fluid biomarkers, and disease progression. Our findings indicate that, in an elderly cohort spanning the entire AD continuum and, to a lesser extent, in non-AD amnestic patients, low FBP retention in the WM was associated with (1) MRI markers of WM degeneration, (2) abnormal AD fluid biomarkers, and (3) increased risk of progressive cognitive decline in early-stage AD. Taken together, our findings add to a growing body of literature linking WM abnormalities in multiple sclerosis and aging with reduced colocalized Aβ PET tracer uptake [19–29] and further highlight the importance of demyelination, assessed with FBP PET, as a marker of disease progression at both preclinical and prodromal stages of AD.

Consistent with previous reports in multiple sclerosis and aging [19–21, 23, 28, 29], we found that, among subjects spanning the AD continuum, FBP retention in WMH regions was lower compared with NAWM. Importantly, these findings are unlikely driven by partial volume effects since we both performed an erosion procedure (which excludes WM voxels affected from spill-out to CSF) and removed small WMH clusters (most affected by spill-in counts from surrounding NAWM); therefore, remaining NAWM spill-in counts into WMH cannot explain the observed lower FBP SUVR in these areas. We also found that FBP uptake in NAWM was associated with FA, a marker of WM integrity, in line with findings from a previous study [44]. Furthermore, we extended this previous knowledge by observing that low FBP retention in NAWM was associated with (1) longitudinal WMH progression, suggesting that FBP is able to capture early demyelinating changes that subsequently progress into WMH, and (2) abnormal plasma NfL levels, indicating that FBP PET may capture myelin loss due to degeneration of large-calibre axons. Overall, our results provide additional evidence supporting the hypothesis that FBP signal in the WM is myelin-dependent and highlights the potential of FBP PET for assessing early WM pathology, though its clinical relevance at the individual level remains to be determined in future dedicated studies.

Another key finding of our study is the observation that FBP-measured demyelination levels progress across the AD continuum, with detectable myelin alterations even at the preclinical stage of AD. This result was further supported by a significant association between FBP-measured demyelination levels and specific CSF biological markers of AD. All these findings resonate with results from previous reports: MRI-based measures of myelin water fraction were found to be associated with CSF Aβ-42 levels in CN individuals [18], and demyelination is a common feature of AD brains at autopsy [15–17]. In this regard, the findings of this study contribute to better understand other degenerative features associated to AD pathology that have been traditionally attributed to age-related concomitant pathologies [55].

We also found that FBP-measured demyelination levels had a relevant impact in the clinical progression of preclinical and prodromal AD participants. These findings expose the clinical relevance of WM pathology in AD and point towards this structure as a potential therapeutic target [55]. Moreover, our findings might also help to increase the positive predictive value of Aβ PET. Since elevated Aβ burden is commonly found in cognitively healthy elderly individuals [56], Aβ PET has been deemed useful mainly for ruling out AD as the underlying cause of a patient’s cognitive impairment [57]. However, through the complementary assessment of WM uptake, Aβ PET might become a clinical tool more closely related to cognitive deficits and short-term progression. Investigations shall be carried out to see as how to best incorporate this new information together with other PET measures of brain amyloidosis [58, 59] in further studies.

The findings in the primary cohort of AD-continuum patients were largely replicated in the secondary cohort of non-AD amnestic patients, though some of the observed associations with longitudinal cognitive decline, CSF p-tau181, and DTI metrics were not statistically significant among these individuals. Multiple complementary factors might explain these differences between AD and non-AD individuals. First, previous evidence with MRI-based measures of myelin integrity indicate that widespread WM demyelination is specifically associated with amyloid pathology in cognitively unimpaired individuals [18], thus suggesting that demyelination might have differential impact on cognition and biomarkers in AD compared with non-AD conditions. Second, the ADNI cohort has been highly preselected to exclude the maximum number of cases with non-AD conditions, in particular with strong exclusion criteria for vascular pathology. Since vascular pathology is a major contributor to WM abnormalities, including demyelination [15], the systematic exclusion of individuals with evident vascular pathology might explain why FBP signal in NAWM is not associated with cognitive decline in the A- MCI group. Third, according to previous reports, a significant fraction of the A- MCI participants actually represent false-positive MCI cases, many of them being diagnosed as cognitively normal during follow-up [60, 61]. The heterogeneity induced by including false-MCI subjects might result in reduced statistical power. Fourth, CSF p-tau181 has been previously shown to be specifically elevated in Alzheimer’s disease and not in other tauopathies; thus one can expect not to find an association with demyelination in non-AD amnestic syndromes [62]. Fifth, sample sizes were lower in amyloid-negative, cognitively impaired groups, which reduce statistical power.

A strength of this study is the relatively large sample of participants covering the entire AD spectrum, with a comprehensive battery of cross-sectional and longitudinal multimodal biomarker data. This work also had several limitations. The ADNI inclusion criteria require participants relatively free of vascular pathology, which might compromise the generalizability of our findings in a community setting. Only a relatively small subset of participants had available DTI scans, which limited our statistical power to conduct more detailed and stage-specific analyses, as well as to draw more robust conclusions about the performance of adjusted SUVRs in comparison to DTI metrics.

In conclusion, our findings extend previous results suggesting that Aβ PET might be used for assessing early WM abnormalities in AD. These WM changes progressed across the AD spectrum and with increasingly abnormal AD-specific biomarkers and further predicted longitudinal AD clinical progression. The findings of the study highlight the clinical relevance of AD-related WM pathology.

## Supplementary Information

Below is the link to the electronic supplementary material.

Supplementary file1 (DOCX 2024 KB)

## Data Availability

All the data used in this study is publicly available at the Laboratory of Neuro Imaging (LONI) server of the Alzheimer’s Disease Neuroimaging Initiative.

## Code availability

Custom code used in this study will be shared to interested investigators upon request.
