# Supplementary material for: 18F-florbetapir PET as a marker of myelin integrity across the Alzheimer’s disease spectrum
Source: Eur J Nucl Med Mol Imaging. 2021 Sep 28;49(4):1242–53. doi: 10.1007/s00259-021-05493-y (PMC8921113; doi:10.1007/s00259-021-05493-y)
Supplement: Supplementary file 1 — Supplementary file1 (DOCX 2024 KB) [file 259_2021_5493_MOESM1_ESM.docx]

**Supplementary Material**

**Supplementary Methods 1.**

The Alzheimer’s Disease Neuroimaging Initiative (ADNI) is an ongoing observational study that was launched in 2003 as a public-private partnership, led by Principal Investigator Michael W. Weiner, MD. ADNI recruits participants at 57 sites in the USA and Canada. The primary goal of ADNI has been to test whether serial MRI, PET, other biological markers, and clinical and neuropsychological assessment can be combined to measure the progression of mild cognitive impairment (MCI) and early Alzheimer’s disease. The study was approved by the Institutional Review Board (IRB) of all participating centres in ADNI. All study participants, or their study partners, provided written informed consent. For the present study, data were obtained from the Laboratory of Neuro Imaging (LONI) database in June 2020.

**Supplementary Methods 2.**

In line with the findings reported in [1,2], we found a positive correlation between FBP SUVR in the WM and cortical FBP SUVR (*r_NAWM_*=0.41, *p*<0.001; *r_WMH_*=0.23, *p<*0.001). As this association has been hypothesized to be driven by binding to diffuse plaques and cerebrovascular amyloid angiopathy [3,4], we aimed to regress out this confounding effect in order to isolate the contributions of myelin integrity to FBP SUVRs. For this, we fitted linear regression models in the CN cohort describing the dependence of FBP SUVRs in NAWM and WMH on cortical FBP SUVR, yielding the following models:

$${SUVR}_{NAWM}=0.67\cdot{SUVR}_{cortical}+1.26$$

$${SUVR}_{WMH}=1.29\cdot{SUVR}_{cortical}-0.36$$

The selection of a linear model relies on the fact that the dependency of cortical SUVR on NAWM SUVR and WMH SUVR does not significantly deviate from linearity across the different study groups, as illustrated below:


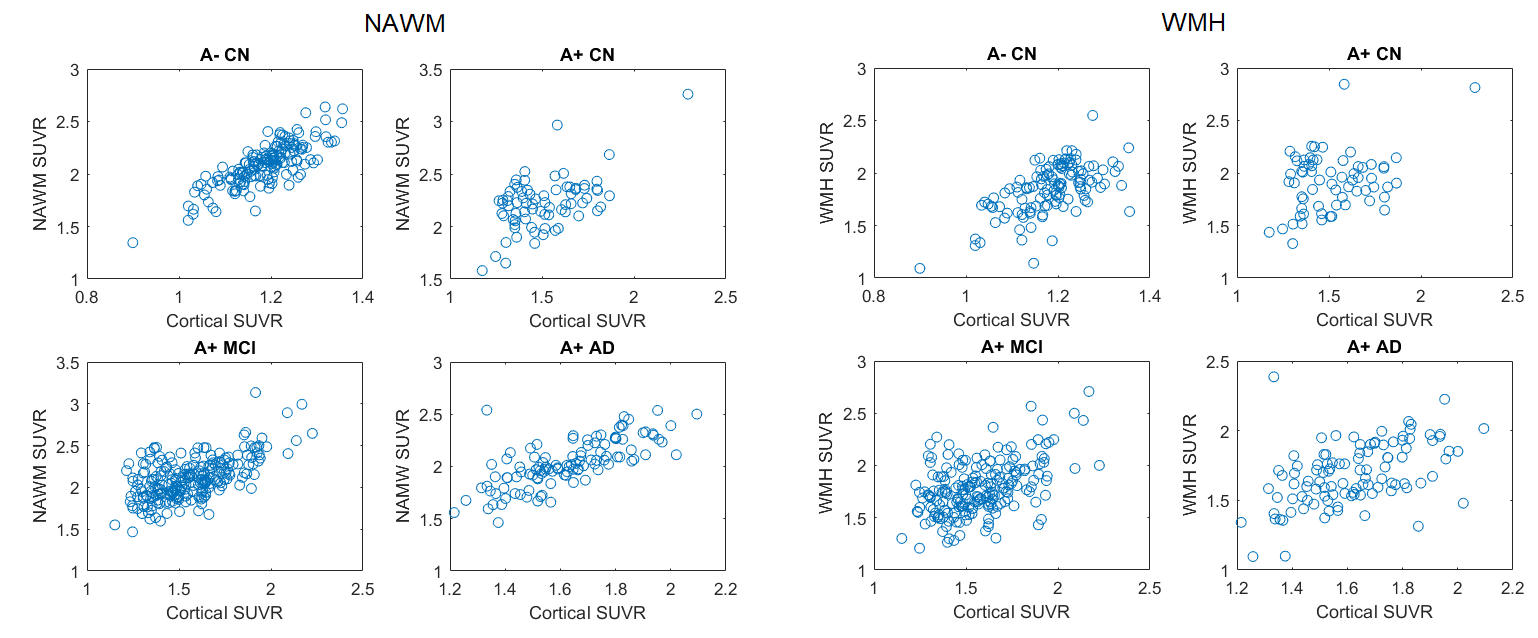


We next regressed-out the dependence on cortical FBP SUVR by subtracting the predicted SUVR (according the above linear models) to each individual SUVR in NAWM or WMH:

$${SUVR}_{NAWM}^{'}={SUVR}_{NAWM}-{SUVR}_{NAWM,pred}$$

$${SUVR}_{WMH}^{'}={SUVR}_{WMH}-{SUVR}_{WMH,pred}$$

Finally, these measures were transformed to z-scores with reference to the ${SUVR}^{'}$ levels of the CN cohort and referred to as “adjusted NAWM (or WMH) SUVR”:

$$adjusted NAWM SUVR= \frac{{SUVR}_{NAWM}^{'}-\mu_{NAWM, CN}}{\sigma_{NAWM, CN}}$$

$$adjusted WMH SUVR= \frac{{SUVR}_{WMH}^{'}-\mu_{WMH, CN}}{\sigma_{WMH, CN}}$$

Where $\mu_{NAWM, CN}$ ($\mu_{WMH, CN})$ stands for the mean levels of the adjusted NAWM (WMH) SUVR in the CN cohort; $\sigma_{NAWM, CN}$ ($\sigma_{WMH, CN})$ stands for the standard deviation of the adjusted NAWM (WMH) SUVR in the CN cohort.


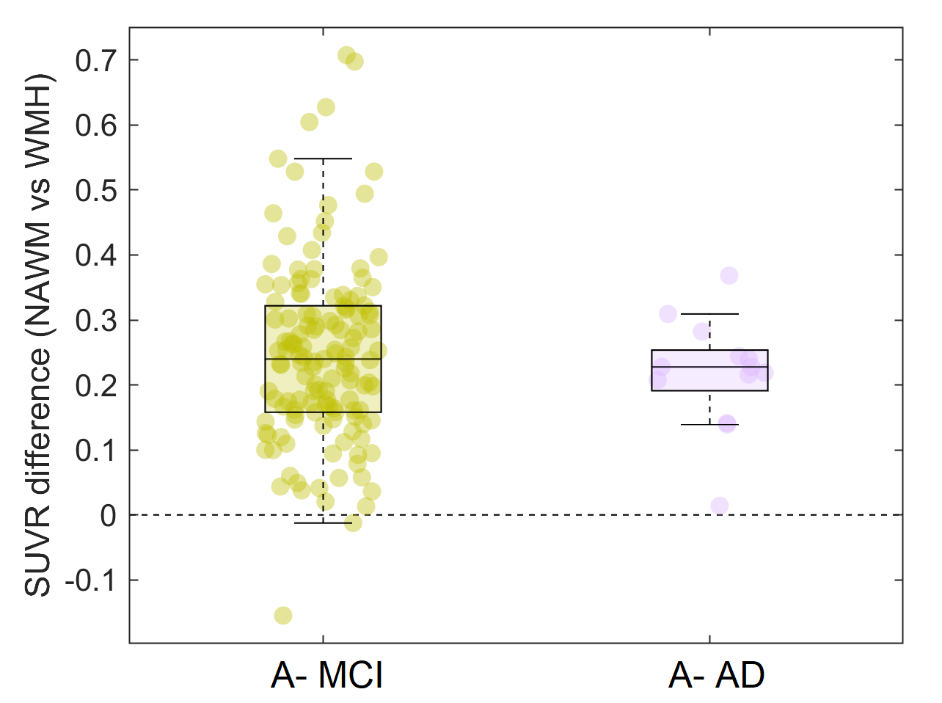
**Supplementary Figure 1.**

**Supplementary Figure 1** - Reduced FBP retention in WMH compared to NAWM in amyloid-negative, cognitively impaired participants. We computed the SUVR difference between NAWM and WMH in amyloid-negative (A-) mild cognitive impairment and A- AD. All the groups showed significantly higher NAWM SUVR compared to WMH SUVR (p<0.001 for A- MCI and p=0.04 for A- AD).

**Supplementary Figure 2.**


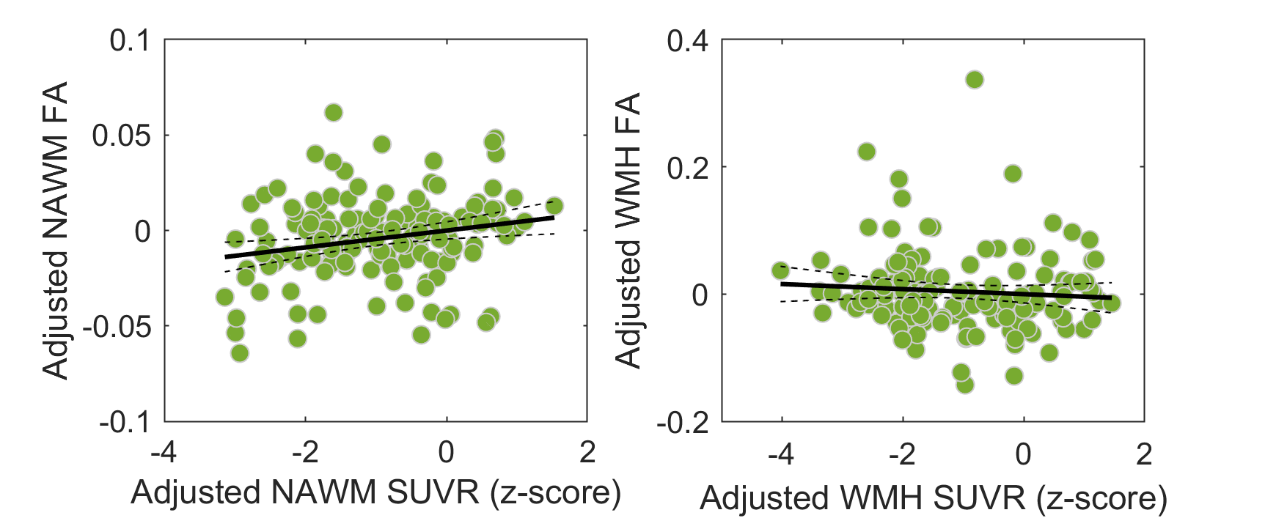


**Supplementary Figure 2** - Association between adjusted SUVR and FA in NAWM and WMH in AD-continuum individuals. Black solid line depicts the regression line. Dashed lines represent 95% confidence intervals. Represented FA values were adjusted for age, sex, and clinical diagnosis.


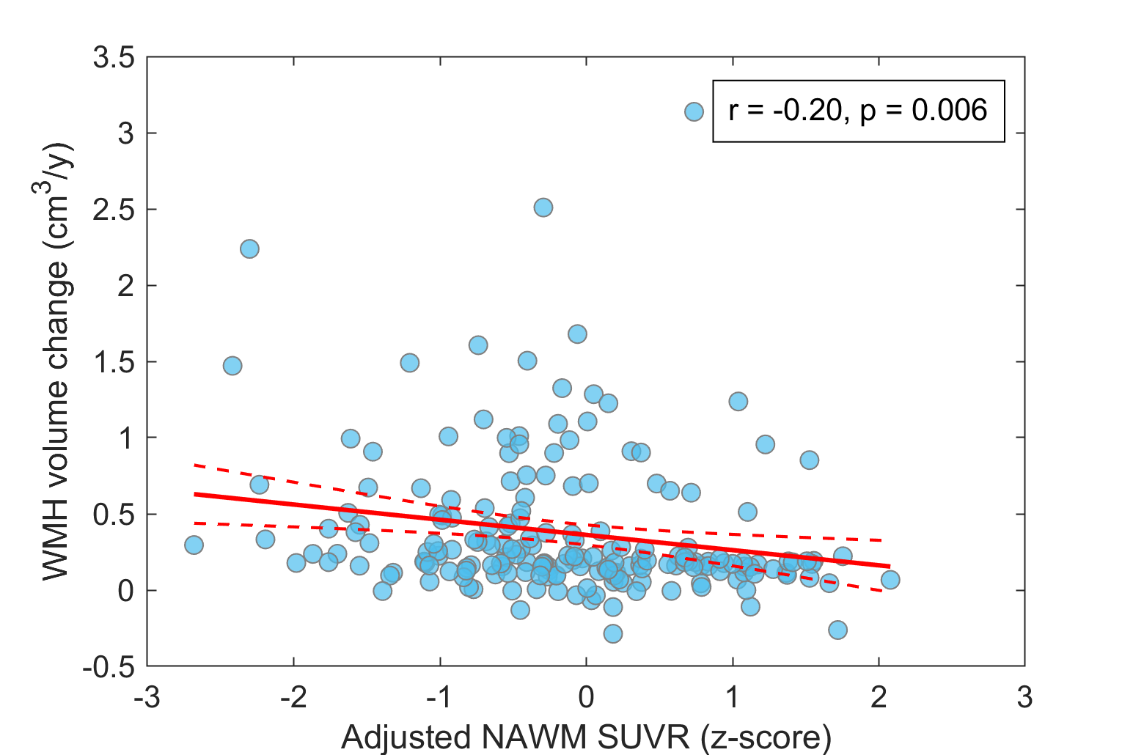
**Supplementary Figure 3.**

**Supplementary Figure 3** - Association between baseline adjusted FBP SUVR in NAWM and longitudinal WMH volume change in amyloid-negative, cognitively impaired participants. Red solid line depicts the regression line. Dashed lines represent 95% confidence intervals. We reported the (unadjusted for covariates) Pearson correlation coefficient describing the correlation between these two variables.

**Supplementary Figure 4.**


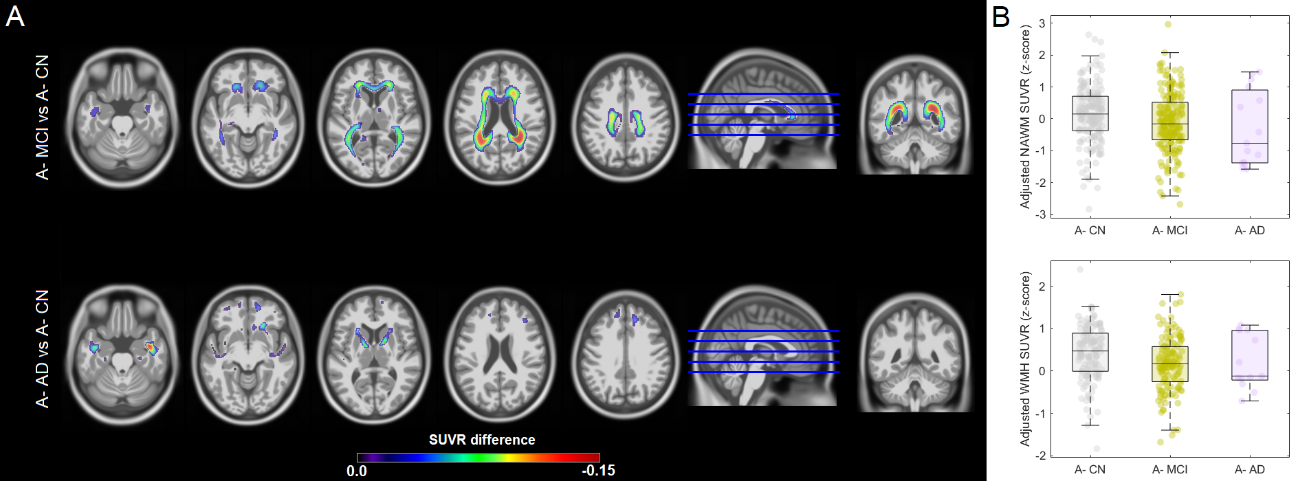


**Supplementary Figure 4** - FBP retention in the WM in amyloid-negative, cognitively impaired participants. A) Voxel-wise analyses, adjusted for age, sex, and cortical FBP SUVR, contrasting SUVR levels in the WM of the different clinical stages (the A- CN cohort was used as the reference). Statistical maps were thresholded using a pFDR<0.05 for A- MCI and an uncorrected p<0.05 for A- AD due to the lower sample size. B) Boxplots describing group-level adjusted SUVR in NAWM and WMH. A- MCI showed significantly lower adjusted NAWM SUVR (p=0.036) and WMH SUVR (p=0.003) but not in A- AD (p>0.56).

**Supplementary Figure 5.**


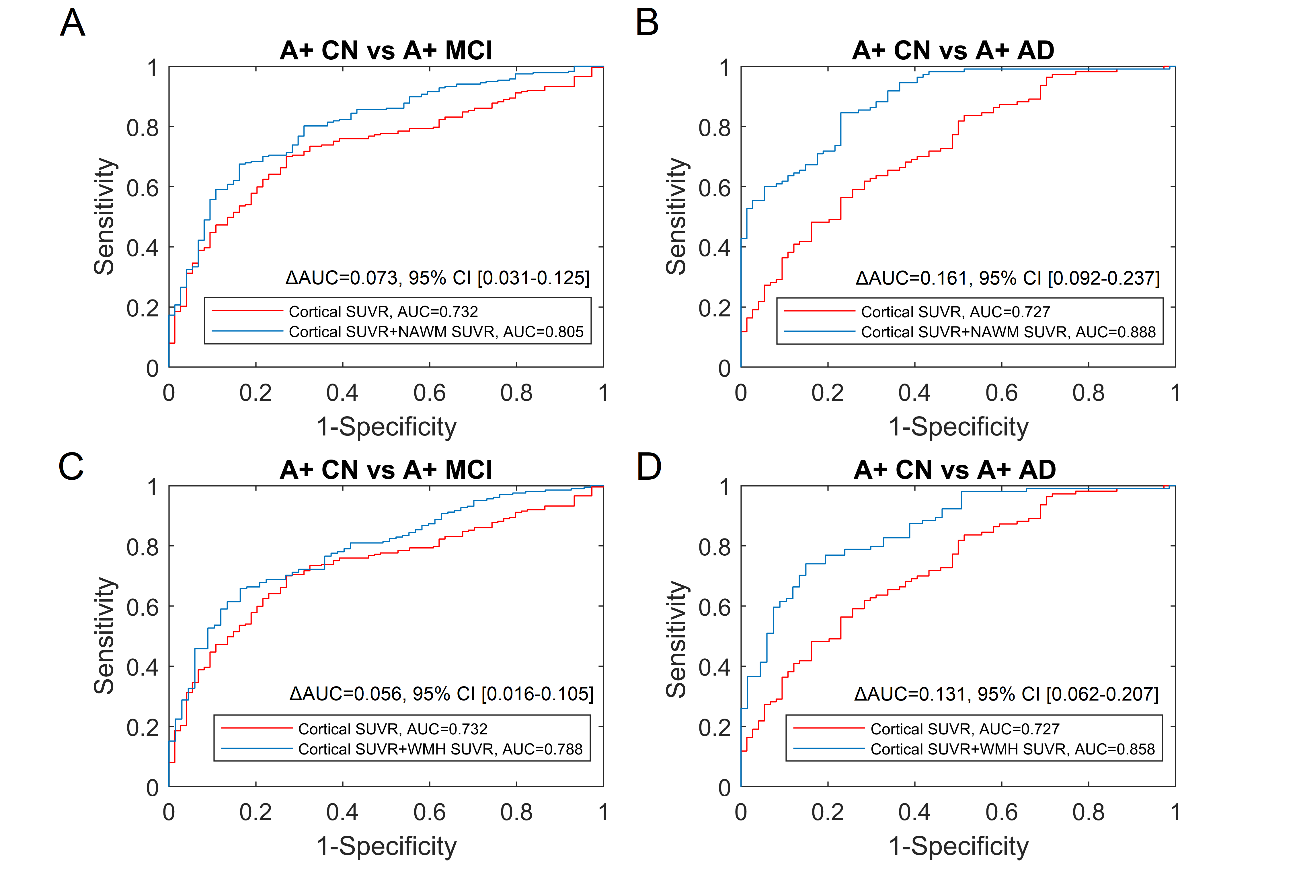


**Supplementary Figure 5** - Receiver operating characteristic (ROC) curve analyses for the discrimination of amyloid-positive cognitively normal individuals vs amyloid-positive mild cognitive impairment and vs amyloid-positive AD dementia. The areas under the ROC curve (AUC) of two age- and sex-adjusted logistic regression were compared: the first model included cortical SUVR, while the second model included both cortical SUVR and adjusted NAWM SUVR (A-B) or adjusted WMH SUVR (C-D). 95% confidence intervals for the difference in AUCs were computed using a 5000-repretition bootstrap procedure.

**Supplementary Figure 6.**

**
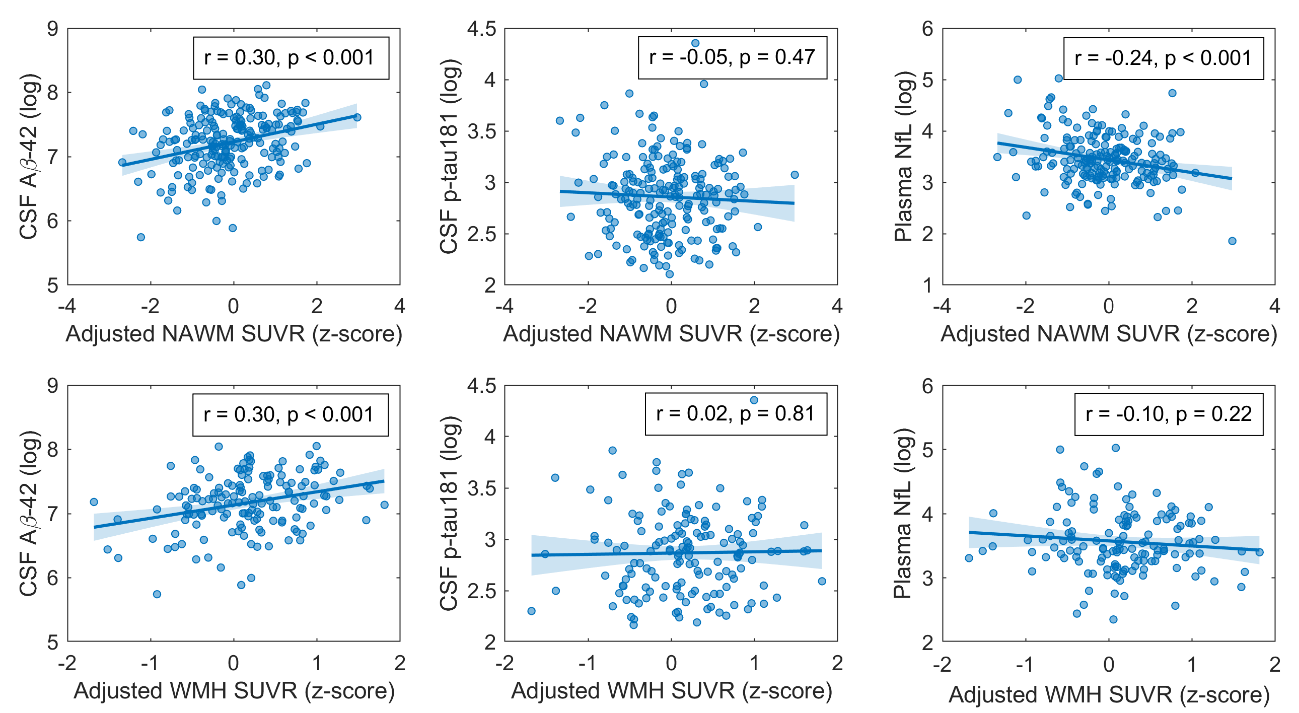
**

**Supplementary Figure 6** - Associations of adjusted SUVR in NAWM and WMH with CSF and plasma biomarkers in amyloid-negative, cognitively impaired participants. We reported the (unadjusted for covariates) Pearson correlation coefficient describing the correlation between the two represented variables. When adjusting for covariates, only the associations with CSF Aβ-42 remained statistically significant (p<0.001).

**Supplementary Figure 7.**


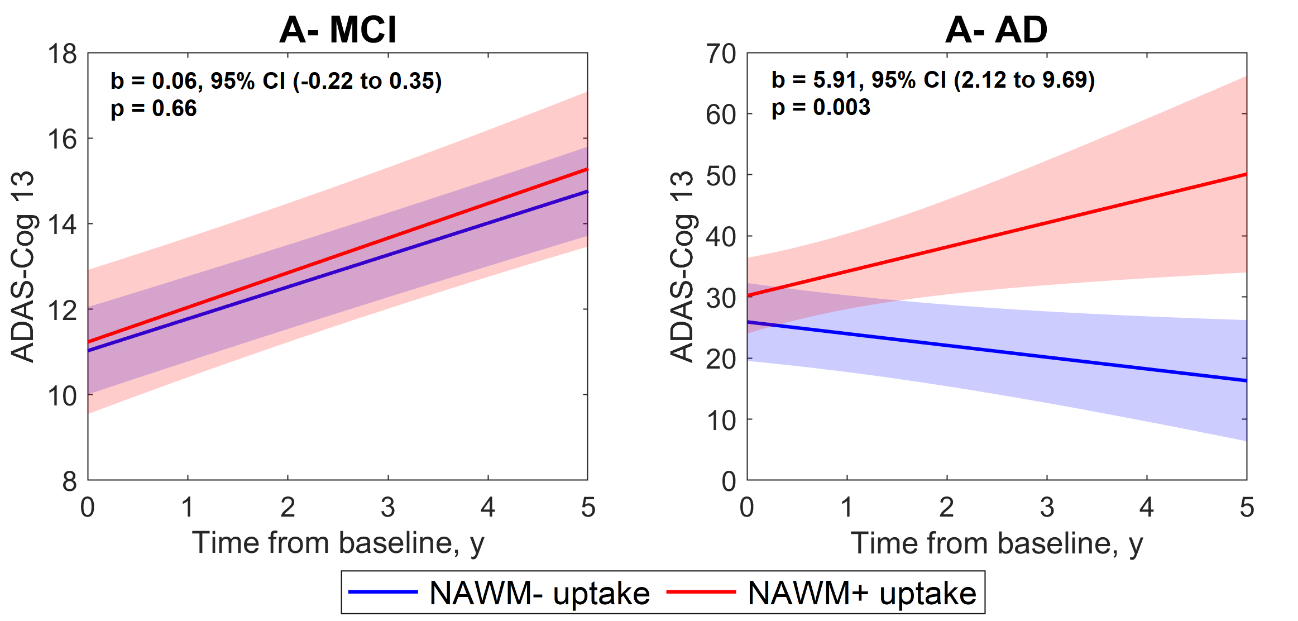


**Supplementary Figure 7** - Associations between dichotomous adjusted FBP SUVR in NAWM and longitudinal clinical decline in amyloid-negative, cognitively impaired participants. Estimated average trajectories of ADAS-Cog 13 for subjects with non-pathological NAWM uptake (NAWM-) (blue line) or pathological NAWM uptake (NAWM+) (red line), stratified in A- MCI and A- AD. Group trajectories were estimated using covariate-unadjusted linear mixed models with subject-specific intercepts. Model coefficients (b) indicate the increase in annual rate of change of ADAS-Cog 13 in the NAWM+ uptake group compared to the NAWM- group.

**Supplementary Table 1.**

*Title:* Demographic and biomarker characteristics of the amyloid-negative, cognitively impaired study participants.

*Legend:* Age and cognitive data (ADAS-Cog 13) were reported as mean (standard deviation). Education years, fluid biomarker levels, and white matter hyperintensity volume were reported as median (range).

|  | **A- MCI** | **A- AD** |
| --- | --- | --- |
| Age, y | 70.2 (7.8) | 77.8 (8.0) |
| Women, n, % | 88, 46% | 1, 7% |
| APOE ε4 carriers, n, % | 48, 25% | 1, 7% |
| Education, y | 16 (11-20) | 16 (12-20) |
| ADAS-Cog 13 | 12.3 (5.4) | 27.9 (7.2) |
| CSF Aβ-42, pg/ml | 1420 (312-3331) | 1381 (402-3139) |
| CSF p-tau181, pg/ml | 17.4 (8.2-52.3) | 25.3 (10.8-77.8) |
| Plasma NfL, pg/ml | 30.0 (6.4-151.8) | 46.8 (22.8-104.9) |
| WMH volume, cm³ | 0.4 (0.0-19.7) | 0.9 (0.0-5.2) |

**Supplementary Table 2.**

*Title:* Head-to-head comparison of the added value of FA versus adjusted SUVR in NAWM and WMH for the discrimination of amyloid-positive cognitively normal individuals vs amyloid-positive mild cognitive impairment and vs amyloid-positive AD dementia.

|  | **Cortical SUVR+**  **NAWM FA** | **Cortical SUVR+**  **adjusted NAWM SUVR** | **Cortical SUVR+**  **WMH FA** | **Cortical SUVR+**  **adjusted**  **WMH SUVR** |
| --- | --- | --- | --- | --- |
| A+ CN vs A+ AD dementia | 0.810 | 0.891 | 0.804 | 0.892 |
| A+ CN vs A+ MCI | 0.821 | 0.787 | 0.830 | 0.810 |

*Legend:* Areas under the ROC curve (AUC) of two age- and sex-adjusted logistic regression were compared: the first model included FA (in NAWM or WMH) and cortical SUVR, while the second model included cortical SUVR and adjusted SUVR (in NAWM or WMH).

**Alzheimer’s Disease Neuroimaging Initiative (ADNI) funders**

Data collection and sharing for this project was funded by the Alzheimer's Disease Neuroimaging Initiative (ADNI) (National Institutes of Health Grant U01 AG024904) and DOD ADNI (Department of Defense award number W81XWH-12-2-0012). ADNI is funded by the National Institute on Aging, the National Institute of Biomedical Imaging and Bioengineering, and through generous contributions from the following: AbbVie, Alzheimer’s Association; Alzheimer’s Drug Discovery Foundation; Araclon Biotech; BioClinica, Inc.; Biogen; Bristol-Myers Squibb Company; CereSpir, Inc.; Cogstate; Eisai Inc.; Elan Pharmaceuticals, Inc.; Eli Lilly and Company; EuroImmun; F. Hoffmann-La Roche Ltd and its affiliated company Genentech, Inc.; Fujirebio; GE Healthcare; IXICO Ltd.; Janssen Alzheimer Immunotherapy Research & Development, LLC.; Johnson & Johnson Pharmaceutical Research & Development LLC.; Lumosity; Lundbeck; Merck & Co., Inc.; Meso Scale Diagnostics, LLC.; NeuroRx Research; Neurotrack Technologies; Novartis Pharmaceuticals Corporation; Pfizer Inc.; Piramal Imaging; Servier; Takeda Pharmaceutical Company; and Transition Therapeutics. The Canadian Institutes of Health Research is providing funds to support ADNI clinical sites in Canada. Private sector contributions are facilitated by the Foundation for the National Institutes of Health (www.fnih.org). The grantee organization is the Northern California Institute for Research and Education, and the study is coordinated by the Alzheimer’s Therapeutic Research Institute at the University of Southern California. ADNI data are disseminated by the Laboratory for Neuro Imaging at the University of Southern California.

**References**

[1] Lowe VJ, Lundt ES, Senjem ML, et al. White Matter Reference Region in PET Studies of (11)C-Pittsburgh Compound B Uptake: Effects of Age and Amyloid-beta Deposition. J Nucl Med. 2018;59:1583-1589.

[2] Zeydan B, Schwarz CG, Lowe VJ, et al. Investigation of white matter PiB uptake as a marker of white matter integrity. Ann Clin Transl Neurol. 2019;6:678-688.

[3] Iwamoto N, Nishiyama E, Ohwada J, Arai H. Distribution of amyloid deposits in the cerebral white matter of the Alzheimer's disease brain: relationship to blood vessels. Acta Neuropathol. 1997;93:334-340.

[4] Lockhart A, Lamb JR, Osredkar T, et al. PIB is a non-specific imaging marker of amyloid-beta (Abeta) peptide-related cerebral amyloidosis. Brain. 2007;130:2607-2615.
